# Circ_0004951 Promotes Pyroptosis of Renal Tubular Cells *via* the NLRP3 Inflammasome in Diabetic Kidney Disease

**DOI:** 10.3389/fmed.2022.828240

**Published:** 2022-06-06

**Authors:** Yulin Wang, Li Ding, Ruiqiang Wang, Yanhong Guo, ZiJun Yang, Lu Yu, LiuWei Wang, Yan Liang, Lin Tang

**Affiliations:** ^1^Department of Nephrology, Zhengzhou University First Affiliated Hospital, Henan, China; ^2^Henan Sheng Zhiyebing Fangzhi Yanjiu Yuan, Henan Institute for Occupational Medicine, The Third People's Hospital of Henan Province, Henan, China

**Keywords:** circ_0004951, NLRP3 inflammasome, pyroptosis, renal tubular cells, diabetic kidney disease

## Abstract

**Background:**

Diabetic kidney disease (DKD) has become the leading cause of chronic kidney disease (CKD) in many countries. Recent studies have shown that circular RNA and pyroptosis play an important role in pathogenesis of DKD.

**Methods:**

We analyzed expression patterns of circRNAs in human kidney biopsy tissues obtained from type 2 DKD (*n* = 9) and nephrectomy (*n* = 9) patients. Next, we cultured human renal tubular epithelial cells (HK2) in high glucose condition and detected circ_0004951, miR-93-5p, NLR Pyrin Domain Containing 3 (NLRP3) inflammasome-related indicators and pyroptosis. Furthermore, we performed Bioinformatics analysis and dual-luciferase reporter assay to analyze the relationship among circ_0004951, miR-93-5p and NLRP3.

**Results:**

Circ_0004951 was significantly upregulated in kidney tissues from DKD patients and HK2 in high glucose condition vs. control. Knockdown of circ_0004951 mediated a significant suppression of HK2 pyroptosis, while results from bioinformatics analysis revealed that circ_0004951 has binding sites with miR-93-5p and miR-93-5p could bind to NLRP3. Results from dual-luciferase reporter assay further corroborated this finding. Finally, observations from rescue experiments showed that down-regulation of miR-93-5p and upregulation of NLRP3 markedly attenuated the anti-pyroptosis and anti-inflammatory effects of circ_0004951 knockdown on HK2.

**Conclusion:**

Circ_0004951 promotes pyroptosis of renal tubular epithelial cells in DKD *via* the miR-93-5p/NLRP3 inflammasome pathway, suggesting its potential for clinical diagnosis and treatment of DKD.

## Introduction

Chronic kidney disease (CKD), which is characterized by high morbidity and mortality rates, is a major public social problem (1). Diabetic kidney disease (DKD) has emerged as the leading cause of CKD in many developed and developing regions. Previous studies have estimated that about 642 million people will suffer from diabetes worldwide by 2040, 30%−40% of whom will develop DKD (2). To date, traditional treatments, such as controlling blood sugar, lowering blood pressure and reducing urinary protein have not been effective in delaying DKD progression to end stage renal disease (ESRD). Therefore, identification of novel biomarkers for pathogenesis and treatment of DKD is imperative to management of the disease. Circular RNAs (circRNAs) are newly discovered non-coding RNAs that lack a 5′ end cap or 3′ end poly(A) tail, which forms a ring structure with covalent bonds (3). CircRNAs are widely and stably distributed among many species, where they regulate gene expression and development of many diseases (4).

Wen et al. (5) found that circACTR2 was upregulated in DKD, with its knockdown mediating a significant suppression of pyroptosis and inflammatory factors release in renal tubular epithelial cells (RTECs). Some research evidences have suggested that circRNAs may play an important role in pyroptosis of RTECs in DKD (6), while others have demonstrated that renal tubulointerstitial inflammation plays an important role in pathogenesis of DKD (7). However, the precise molecular mechanisms underlying inflammatory process remain unclear. Numerous scholars have focused on pyroptosis, in recent years, with substantial interest directed on innate immune inflammation (8). A variety of risk-related molecules, such as hyperglycemia, fatty acids, oxidative stress, and advanced glycation products, among others, can be recognized by related intracellular pattern recognition receptors (PRRs), activate inflammasomes, induce pyroptosis and lead to cell damage (9). Researchers have hypothesized that inhibiting expression of inflammatory factors and regulating pyroptosis could be a new strategy for prevention and treatment of diabetes and some chronic diseases. Notably, the NLR Pyrin Domain Containing 3 (NLRP3) inflammasome is the most extensively and comprehensively studied inflammasome. Accumulating evidence has shown that activation of the NLRP3 inflammasome and pyroptosis not only play a key role in development of renal tubulointerstitial inflammation but also pathogenesis of DKD (8, 10–12). Results from a previous study demonstrated that high glucose-induced epithelial-to-mesenchymal transition (EMT) was effectively alleviated by NLRP3 knockdown in human RTECs (13). In a DKD mouse model, NLRP3 was remarkable upregulated in the RTECs to induce inflammation (14).

We screened the differentially expressed circRNAs between DKD and matched adjacent normal renal tissues by high-throughput sequencing, and found that circ_0004951 was significantly upregulated in renal tissues of DKD patients. Circ_0004951 in HK2 cultured under high glucose (HG) condition was also significantly upregulated vs. normal glucose (NG) condition.Then we prospected for miRNAs that can bind to circ_0004951 and NLRP3 by screening Circbank and TargetScan databases, and found that circ_0004951 has target sites for miR-93-5p. In addition, down-regulation of miR-93-5p promoted proliferation, migration and fibrosis of high glucose-induced mouse mesangial cells, podocyte and RTECs (15–18), suggesting that miR-93-5p plays a role in pathogenesis of DKD. In addition, we used TargetScan to predict that miR-93-5p had target sites for NLRP3. Therefore, we hypothesized that circ_0004951 might regulate pyroptosis of RTECs in DKD through the miR-93-5p/NLRP3 axis.

## Patients and Methods

### Patients and Samples

Human kidney biopsy tissues were obtained from patients with type 2 DKD (*n* = 9) from the Department of Nephrology in The First Affiliated Hospital of Zhengzhou University. We also obtained normal kidney tissues from nephrectomies of renal hamartoma (*n* = 9) as controls. A summary of the patients' clinical data is shown in Table 1. Part of the kidney tissues were used for pathological diagnosis, while the rest were used for analysis of circRNA expression. We first performed differential circRNA expression analysis in three cases of DKD kidney alongside three cases of normal kidney tissues by Biomarker Technologies (Beijing, China). DKD was diagnosed according to previously reported pathological classification standards (19).

**Table 1:** The sequences of the PCR primer pairs used in this study.

**Gene**	**Sequences**
Circ_0004951	Forward, 5′-CTTCACCCACGAATCAAGCAG-3′
	Reverse, 5′-ACATCCTGGAAGGCATCTGTG-3′
TRPM7	Forward, 5′-TGCTGTTTCCCCTCCAGAAC-3′
	Reverse, 5′-GTGGCTTTTGCAACTTGGCT-3′
miR-93-5p	Forward, 5′-AGGCCCAAAGTGCTGTTCGT-3′
	Reverse, 5′-GTGCAGGGTCCGAGG-3′
NLRP3	Forward, 5′-AGAGCCCCGTGAGTCCCATTAAG-3′
	Reverse, 5′-CGCCCAGTCCAACATCATCTTCC-3′
GAPDH	Forward, 5′-TCAACAGCGACACCCACTCC-3′
	Reverse, 5′-TGAGGTCCACCACCCTGTTG-3′

### Cell Culture and Treatments

Human renal tubular epithelial cells (HK2) were obtained from The Cell Bank of Type Culture Collection of The Chinese Academy of Sciences. The cells were cultured in DMEM/F12 (Invitrogen; Thermo Fisher Scientific, Inc.) supplemented with 10% fetal bovine serum (Invitrogen; Thermo Fisher Scientific, Inc.), and 1% penicillin-streptomycin solution (Invitrogen; Thermo Fisher Scientific, Inc.), and incubated under conditions of 5% CO_2_ and 37°C. Medium for the control group contained 5.6 mmol/L d-glucose (normal glucose, NG), cells in the experimental group were incubated in medium containing 30 mmol/L d-glucose (high glucose, HG) for 48 h, while those in the negative control group were cultured in medium comprising 5.6 mmol/L d-glucose with mannitol 24.4 mmol/L (hyperosmotic, HO) (5, 20).

### Cell Transfection

To knockdown circ_0004951, we transfected HK-2 cells using small interference RNAs from Geneseed Co., Ltd. (Guangzhou, China). All transfections were according to the manufacturer's protocol. The cells were then exposed to NG or HG for 48 h. MiR-93-5p mimic/inhibitor and overexpression vector of NLRP3, with their corresponding controls, were all purchased from Ribobio Co., Ltd. (Guangzhou, China).

### Enzyme-Linked Immunosorbent Assay

Levels of IL-1β and IL-18 in cell culture supernatants were determined using Enzyme-Linked Immunosorbent Assay (ELISA) kits (Genmed Scientifics, Inc.), according to the manufacturer's instructions.

### Quantitative Real-Time Polymerase Chain Reaction (qRT-PCR)

Total RNA was extracted from kidney tissues and HK2 cells using RNAiso Plus kits (TaKaRa, Dalian, China), according to the manufacturer's instructions. The RNA was quantified, then reverse-transcribed into cDNA using a Reverse Transcription kit (TaKaRa). Equal concentrations of the cDNA were subjected to qRT-PCR using the SYBR Premix Ex Taq II (TaKaRa), and performed on a CFX Realtime PCR system (Bio-Rad, Hercules, CA, USA), targeting specific genes whose primers are outlined in Table 2 (6, 15). Relative gene expression was calculated using the 2^−ΔΔCt^ method, and compared to that of GAPDH used as an internal amplification control.

**Table 2:** Clinical data of patients.

	**Age(years)**	**Sex**	**24hTP (g)**	**Hb (g/L)**	**HbA1C (%)**	**Scr (μmol/L)**	**Alb (g/L)**	**eGFR (ml/min/1.73 m^**2**^)**
Patient 1*	38	Male	1.03	153	8.4	69	48.7	114.475
Patient 2*	45	Female	4.19	80	6.3	91	29.4	68.578
Patient 3*	53	Female	2.81	127	7.3	126	39.8	55.734
Patient 4	40	Male	0.83	151	6.7	66	45	114.959
Patient 5	46	Male	10.12	110	5.6	167	23.5	41.645
Patient 6	55	Male	7.31	98	8.3	202	30.1	31.060
Patient 7	46	Male	2.7	132	7.1	59	40.7	115.412
Patient 8	54	Female	3.51	94	8.2	85	28.1	67.133
Patient 9	49	Female	6.34	86	5.5	435	27.5	9.659
Control 1*	48	Male	0.14	122	5.1	101	38.8	70.306
Control 2*	53	Female	0.06	127	4.6	77	42.5	97.110
Control 3*	45	Male	0.11	146	5.9	70	42.3	107.581
Control 4	59	Male	0.16	162	4.7	84	46.3	87.239
Control 5	62	Male	0.04	135	4.2	63	34.7	120.951
Control 6	58	Female	0.18	118	5.3	58	35.6	97.875
Control 7	66	Male	0.12	137	5.7	76	39.7	93.736
Control 8	68	Female	0.19	108	5.1	69	32.4	78.294
Control 9	57	Female	0.03	119	4.5	70	40.2	83.125

*The kidney tissues of these three pairs of patients were subjected to circRNA high-throughput sequencing.

*24hTP, 24h urine total protein; Hb, hemoglobin; HbA1C, glycosylated hemoglobin; Scr, serum creatinine; Alb, serum albumin; eGFR, estimated glomerular filtration rate*.

### Western Blot Assay

Total proteins were extracted from HK2 cells on ice using a high-efficiency lysis buffer (Solarbio, Beijing, China). Protein concentration was determined using the BCA kit (Beyotime, Shanghai, China), 50 μg from each sample separated *via* SDS-PAGE (10% gel), then transferred onto polyvinylidene fluoride membranes. The membranes were incubated overnight with primary antibodies against NLRP3, IL-1β, and IL-18, with GAPDH as an internal control. Next, the membranes were incubated with corresponding secondary antibodies for 2 h. Signals were visualized using an chemiluminescence imaging system (Amersham Imager 680, cytiva, America), and protein expression analyzed using ImageJ software (National Institutes of Health Software, Bethesda, Maryland). The following antibodies were used for protein detection: Anti-NLRP3 (Abcam, ab263899; 1:1,000), anti-IL-1β (Abcam, ab216995; 1:500), anti-IL-18(Abcam, ab207324; 1:500).

### Cell Counting Assay (CCK-8)

Cells at the logarithmic growth phase were plated in 96-well plate (100 μl of culture medium containing 1 × 10^4^ cells per well, then incubated for 48 h under different environments (NG, HG, and HO). Next, 10 μl of CCK-8 reagent (Dojindo, Tokyo, Japan) was added to each well, followed by 4–5 h incubation. Absorbance for each experiment was detected using a Microplate reader (Thermo Fisher Scientific, Waltham, MA, USA), at 450 nm. Finally, the proliferation inhibition rate of HK2 cell was calculated under different environments. Each experiment was repeated three times, and the average value was taken to calculate the proliferation inhibition rate.

### Bioinformatics Analysis

Target genes of circRNA were predicted using the online database Circbank (http://www.circbank.cn/searchCirc.html), while the interaction between miR-93-5p and NLRP3 was determined using TargetScan (version 7.2, http://www.targetscan.org/vert_72/).

### Dual-Luciferase Reporter Assay

The 3′UTR region of NLRP3 or circRNA sequences were cloned downstream of the reporter gene Luciferase in the vector to construct pRL-TK plasmid (Promega, Madison, WI, USA). These were then transfected into cells, and changes in reporter gene expression detected by comparing the overexpressed or interfering miRNAs (with firefly luciferase used as the reporter gene and Renilla luciferase was used as the internal reference gene) to quantitatively reflect the inhibition effect of miR-93-5p on the target gene.

### Lactate Dehydrogenase Release Assay

Lactate dehydrogenase (LDH) released into the supernatants was detected, in order to evaluate pyroptosis of HK2. Briefly, 10 μl of supernatants was subjected to LDH detection using the LDH assay kit (Jiancheng, Nanjing, China) according to the manufacturer's instructions (21).

### Caspase-1 Activity Assay

HK2 cells were collected after 48 h of culture under different environments, then subjected to detection of caspase-1 activity using the Caspase 1 Activity Assay Kit (C1101; Beyotime Biotechnology), according to the manufacturer's instructions.

### Statistical Analysis

Statistical analyses were performed using GraphPad Prism version 8.3.0 (GraphPad Software, USA), and all data presented as means ± standard deviations (SD). Differences between groups were determined using an unpaired Student's *t*-test whereas those among multiple groups were analyzed using One-way analysis of variance (ANOVA), followed by Bonferroni *t*-tests. Data followed by *p* < 0.05 were considered statistically significant.

## Results

### Profile of CircRNA Expression in DKD Patients

**Figure 1:**
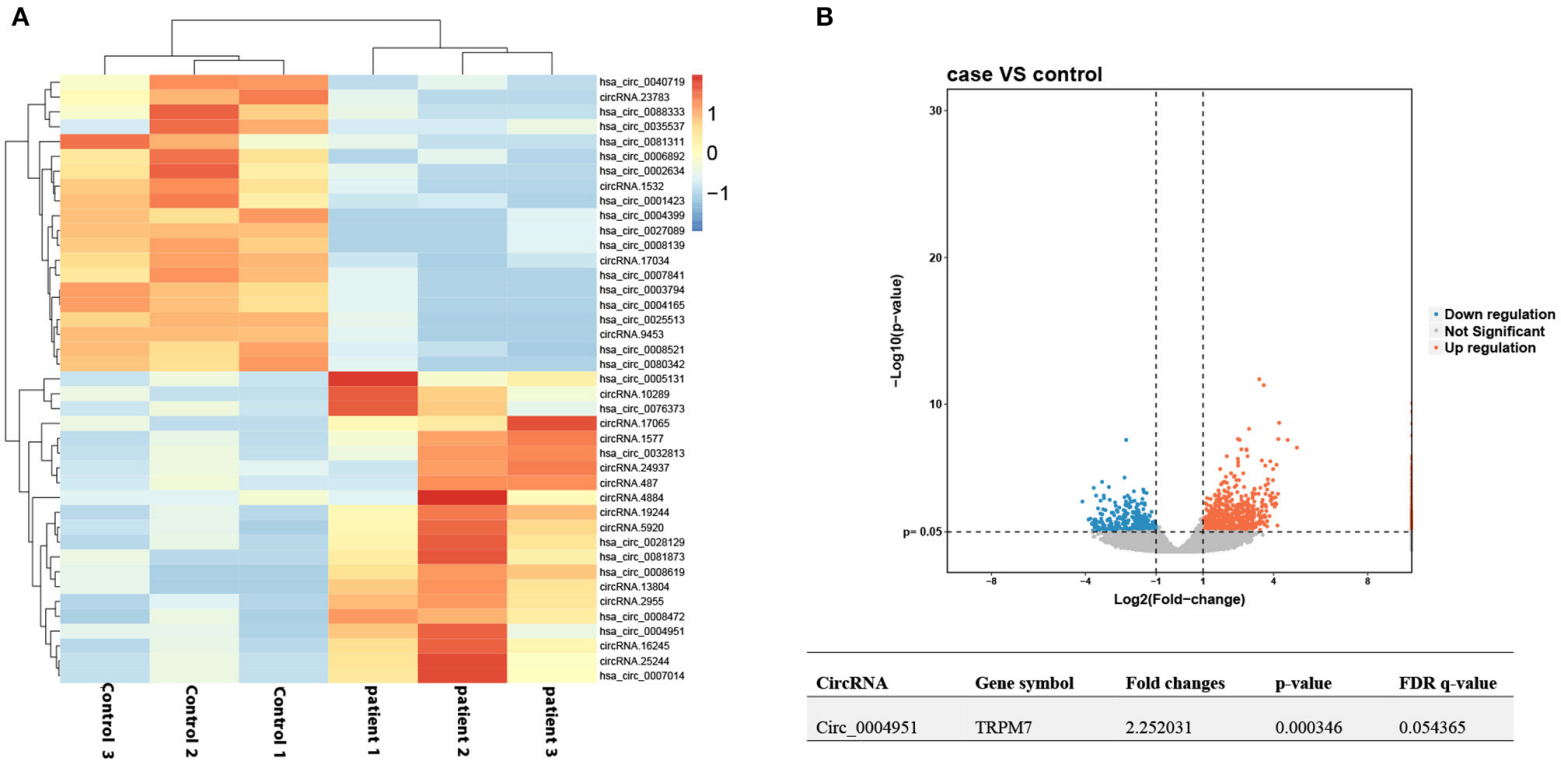
Profile of circ_00004951 expression in kidney tissues of DKD patients. **(A)** Heat maps and **(B)** volcano plots.

We applied high-throughput sequencing to detect expression patterns of circRNAs in kidney tissues of three DKD patients alongside three normal kidney tissues. Microarray data of aberrantly expressed circRNAs are presented using a heat map and volcano plots (Figures 1A,B). Among the candidate circRNAs, we focused on a significantly upregulated RNA, namely circTRPM7 (circBase ID: hsa_circ_0004951, http://www.circbase.org/).

### Circ_0004951 Is Upregulated in Renal Tissues of DKD Patients

**Figure 2:**
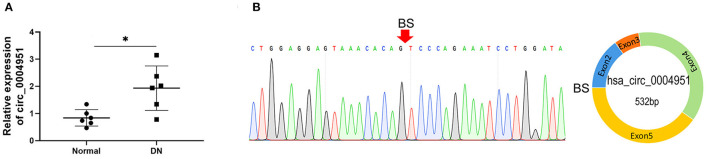
Upregulation of circ_0004951 in renal tissue from DKD patients. qRT-PCR results showing levels of circ_0004951 expression in renal tissues from DKD patients (*n* = 6) and normal renal tissue (*n* = 6) **(A)**. Presence of BS sites in kidney tissue RNA from DKD patients following amplification and sequencing with divergent primers **(B)**. DKD, diabetic kidney disease; qRT-PCR, quantitative real-time polymerase chain reaction. *p < 0.05 vs. normal tissues.

Circ_0004951 was significantly upregulated in renal tissue samples of DKD patients (*n* = 6) relative to normal renal tissues (*n* = 6; Figure 2A). Amplification of the RNA from renal tissue of patients with DKD using divergent primers revealed presence of back splicing (BS) sites (Figure 2B). Since circ_0004951 is formed by circularization of the second, third, fourth and fifth exons of TRPM7, divergent primer amplification showed the reverse binding sequence between the end of the fifth exon and the second start, confirming the sub-circularity RNA is present (Figure 2B).

### High Glucose Induced Pyroptosis Was Accompanied by an Increase in Circ_0004951 Level

**Figure 3:**
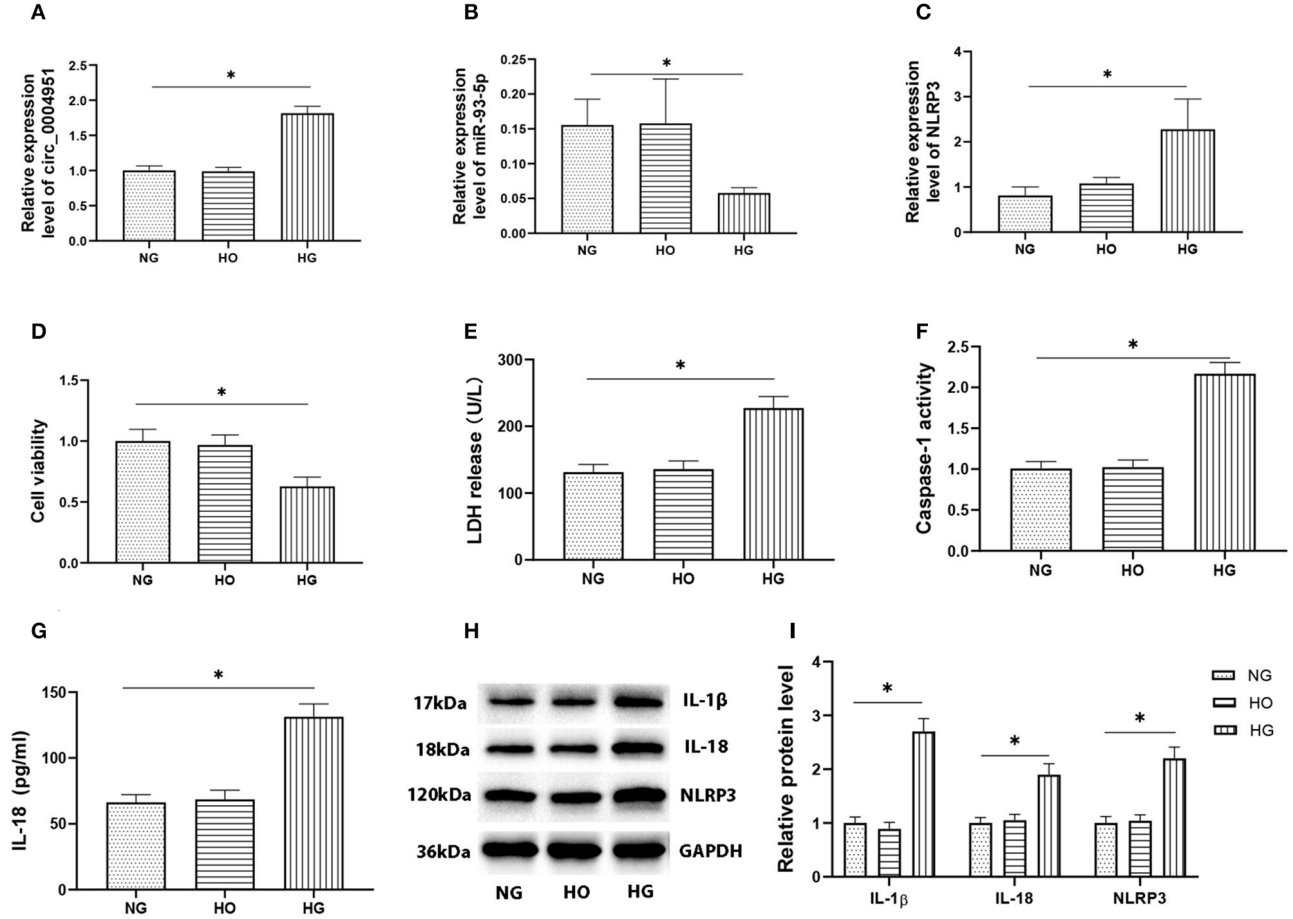
Effect of HG on expression of circ_0004951 and pyroptosis of HK2 cells. qRT-PCR results showing that HG mediated a marked upregulation of circ_0004951 and NLRP3 with a downregulation of miR-93-5p **(A–C)**. CCK-8 assay results showing that HG suppressed HK2 cell viability compared to NG or HO groups **(D)**. HG increased LDH release **(E)**. HG increased the caspase-1 activity **(F)**. ELISA results showing that HG upregulated IL-1β **(G)** and IL-18 **(H)** expression. Western blots showing that HG upregulated IL-1β, IL-18 and NLRP3 expression **(I)**. HG, high glucose; NG, normal glucose (control); HO, hyperosmotic; HK2, human renal tubular epithelial cell line; qRT-PCR, quantitative real-time polymerase chain reaction; CCK-8, cell counting kit-8; LDH, lactate dehydrogenase; NLRP3, NLR pyrin domain containing 3. Values are expressed as mean ± SD. *p < 0.05 vs. control.

Results from qRT-PCR, performed to analyze expression of circ_0004951, miR-93-5p and NLRP3 in HK2 cultured under HG, NG, and HO conditions, revealed the significant changes in HG group (Figures 3A–C). At the same time, CCK8 assay results showed that HG treatment remarkably suppressed cell viability compared to either NG or HO groups (Figure 3D). Moreover, HG mediated an increase in LDH release into the cell culture medium (Figure 3E), the activity of caspase-1 (Figure 3F) and the expression of IL-1β, IL-18 and NLRP3 (Figures 3G–I). Collectively, these results suggested that HG might induce RTECs pyroptosis accompanied by upregulation of circ_0004951.

### Circ_0004951 Directly Binds miR-93-5p to Disinhibit NLRP3 Expression

**Figure 4:**
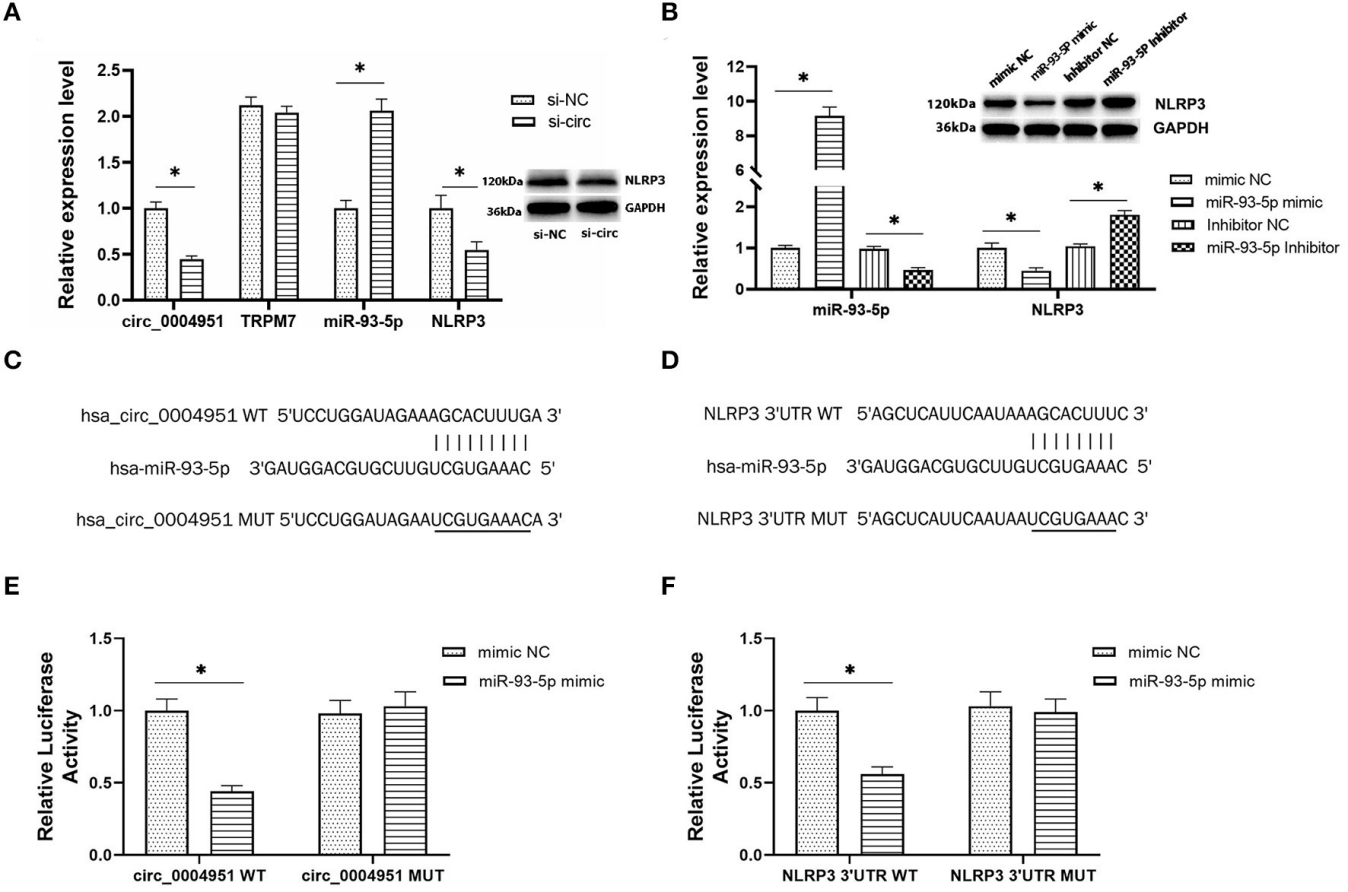
The regulatory relationship between circ_0004951, miR-93-5p and NLRP3. The expression of circ_0004951, parental gene TRPM7, miR-93-5p and NLRP3 after knockdown of circ_0004951 **(A)**. MiR-93-5p mimic and miR-93-5p inhibitor upregulated and downregulated expression of miR-93-5p and NLRP3, respectively **(B)**. Bioinformatics analysis showed the adsorption and binding sites for miR-93-5p in circ_0004951 **(C)**, and seed region sites of NLRP3 3′UTR bound to miR-93-5p **(D)**. Luciferase reporter assay results showing relative luciferase activities after co-transfection of construct containing wt or mut of circ_0004951 and miR-93-5p mimics or inhibitors **(E)**. Luciferase reporter assay results showing luciferase activities after co-transfection with the miR-93-5p mimic containing either wt or mut of NLRP3 **(F)**. NLRP3, NLR pyrin domain containing 3; HK2, human renal tubular epithelial cell line; 3′-UTR, 3′-untranslated region. Values are expressed as mean ± SD. *p < 0.05 vs. control.

According to online database Circbank, there are 53 miRNAs that have binding sites with circ_0004951, and 2 of these miRNAs have binding sites with NLRP3, namely miR-93-5p and miR-17-5p. At the time of our project, the study on miR-93-5p in renal tubulointerstitial fibrosis in DKD has been reported (17, 18), so we chose miR-93-5p. To explore the regulatory relationship between circ_0004951, miR-93-5p and NLRP3, we first transfected si-circ_0004951(si-circ) into HK2, then performed qRT-PCR to verify efficiency of knockdown (Figure 4A). Results showed that knocking down circ_0004951 not only significantly upregulated miR-93-5p in HK2 (Figure 4A), but also markedly downregulated NLRP3 in HK2 cells (Figure 4A). Since TRPM7 also has an impact on NLRP3 inflammasome activation and cell death, we detected the expression of TRPM7 mRNA in HK2 after using circ_0004951 siRNA to ensure that the effects of knockdown of circ_0004951 merely through affecting circ_0004951 but not through influencing TRPM7 expression (Figure 4A). These results suggested that circ_0004951 negatively and positively regulates expression of miR-93-5p and NLRP3, respectively. Next, we transfected HK2 with miR-93-5p mimic and miR-93-5p inhibitor to upregulate and downregulate miR-93-5p expression, respectively (Figure 4B). We found that upregulating miR-93-5p mediated a decrease in NLRP3 levels, while downregulating miR-93-5p upregulated NLRP3 (Figure 4B). These findings suggested that miR-93-5p negatively regulated the expression of NLRP3. Results from bioinformatics analysis demonstrated that circ_0004951 had adsorption and binding sites for miR-93-5p, and its expression might be negatively regulated by molecular sponge adsorption (Figure 4C). This prediction was then verified by dual-luciferase reporter assay (Figure 4E). Targetscan also predicted binding sites between miR-93-5p and NLRP3, with the seed region site found to negatively regulate its expression by binding to its 3′UTR (Figure 4D). Results from dual-luciferase reporter assay revealed that miR-93-5p could be adsorbed and bound to the predicted site of NLRP3 3′UTR, thereby downregulating NLRP3 expression (Figure 4F). Collectively, these results indicated that circ_0004951 promotes pyroptosis of RTECs by upregulating NLRP3 expression *via* miR-93-5p.

### Knockdown of Circ_0004951 Suppressed HG-Induced Pyroptosis *via* the miR-93-5p/NLRP3 Inflammasome Axis

**Figure 5:**
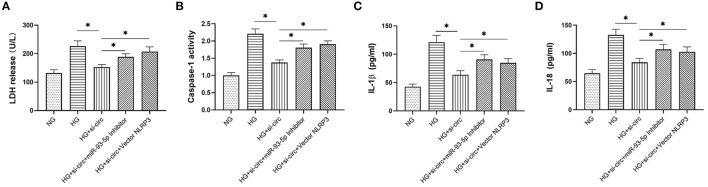
The circ_0004951/miR-93-5p/ NLRP3 axis regulates HG-induced pyroptosis in HK2. LDH release in each group **(A)**. Caspase-1 activity in each group **(B)**. ELISA results showing expression levels of IL-1β and IL-18 in each group **(C,D)**. HG, high glucose; HK2, human renal tubular epithelial cell line; LDH, lactate dehydrogenase. Values are expressed as mean ± SD. *p < 0.05 vs. control.

**Figure 6:**
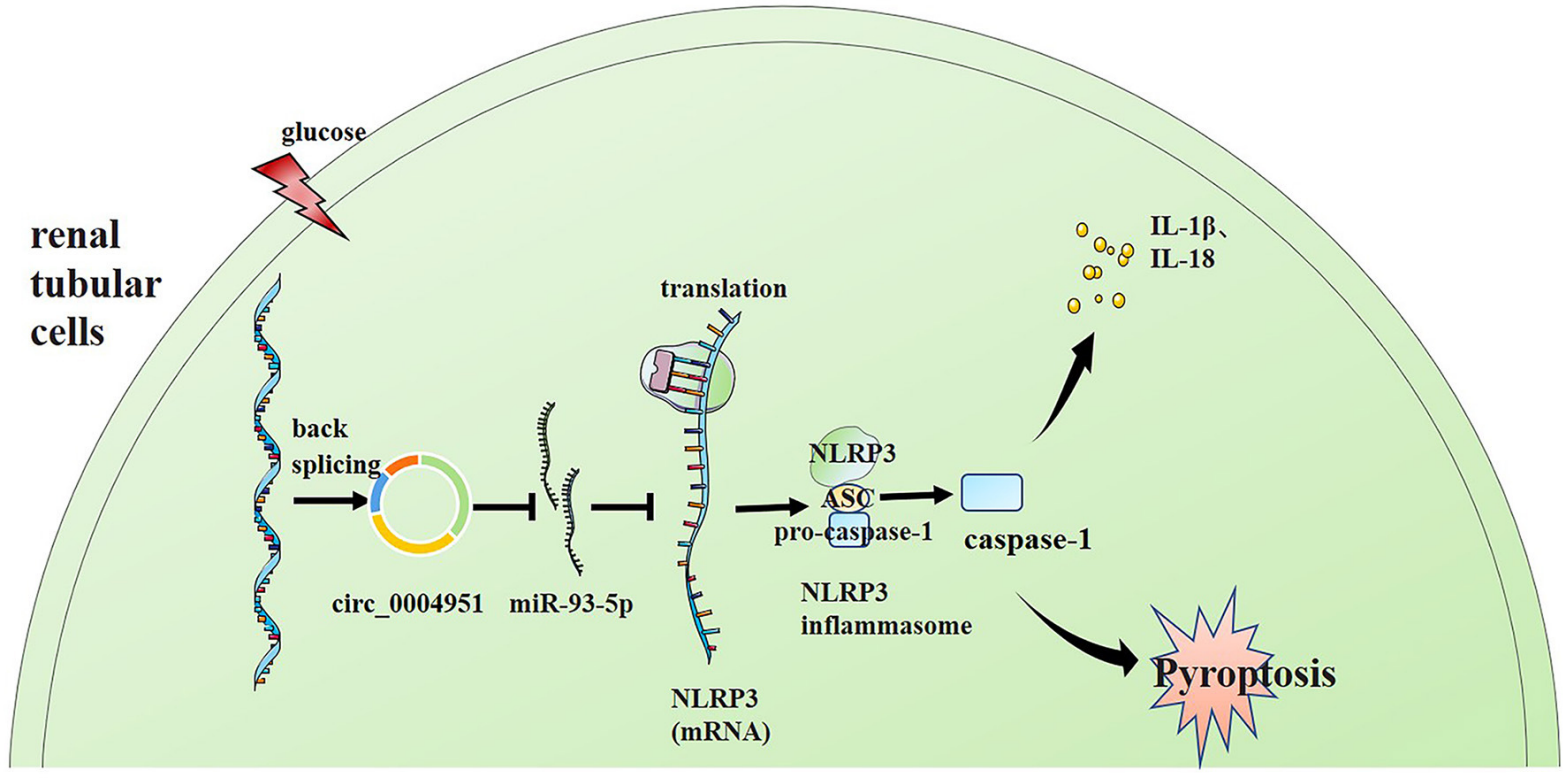
Schematic representation of the proposed molecular mechanism showing that pyroptosis of renal tubular cells occurs *via* the “circ_0004951/miR-93-5p/NLRP3 inflammasome” axis in DKD. DKD, diabetic kidney disease.

Finally, we explored whether miR-93-5p inhibition could partly reverse the effects induced by si-circ_0004951 (si-circ) in HG-induced HK-2 cells *via* NLRP3. Firstly, we analyzed the effect of knocking down circ_0004951 on HK2 cells under HG environment and found that circ_0004951 knockdown mediated a decrease in LDH release and the increase of pyroptosis related indicators (caspase-1, IL-1β and IL-18) induced by HG. Next, we transfected the miR-93-5p inhibitor into HK2 cells and found that it markedly attenuated the inhibitory effect of knocking down circ_0004951 on LDH release and pyroptosis induced by HG. Finally, we transfected NLRP3 overexpression vector (vector NLRP3) into HK2 cells, after knocking down circ_0004951, and found that NLRP3 overexpression significantly attenuated the inhibitory effect of circ_0004951 knockdown on LDH leakage and pyroptosis induced by HG (Figure 5). Taking the regulatory effect of circ_0004951/miR-93-5p axis on NLRP3 into consideration, we concluded that circ_0004951 promotes pyroptosis of renal tubular epithelial cells in DKD by sponging miR-93-5p to induce activity of the NLRP3 inflammasome (Figure 6).

## Discussion

The high incidence of diabetes has negatively impacted public health, worldwide. The latest data, released by the International Diabetes Federation, shows diabetes has a global prevalence rate of 9.3%, with 463 million people reportedly having with diabetes in 2019 alone. By country, China has 116.4 million diabetic patients, accounting for about 1/4 of the global patients (22). DKD is a serious diabetes complication and one of the main causes of ESRD (2). The latest annual data, released by China Kidney Disease Network (CK-NET) in 2020, shows that DKD accounts for about 26.7% of all CKD patients hospitalized in China, and it is the first cause of ESRD in hospitalized patients (23). DKD not only seriously affects patients' quality of life, but also brings a huge economic burden on families and society. To date, however, no effective treatment therapy has been developed.

Although most previous studies on DKD have mainly focused on glomerular lesions, recent evidences have shown that the role of renal tubular injury cannot be ignored (24). Tubular injury is an early indicator of DKD pathology, and may predict as well as participate in its progression (25). Previous studies have revealed that existence of renal tubular damage even when the urinary albumin excretion rate of diabetic patients is normal, indicating that renal tubular disease may play an important role in the occurrence of DKD (26). Brezniceanu et al. (27) also found that there may be RTECs apoptosis and tubular atrophy during early stages of DKD development, especially the proximal renal tubule. Therefore, investigating DKD renal tubular damage may provide new targets for treatment of DKD.

Renal tubular damage in DKD mainly includes apoptosis, EMT, and inflammatory cell infiltration around the tubules. However, recent studies have suggested that pyroptosis also plays an important role in renal tubular injury during DKD development. Pyroptosis is a programmed process that involves cell self-destruction mediated by caspase-1 activation. Previous studies have shown that mechanism and characteristics underlying caspase-1 dependent cell death are different from those that regulate apoptosis. Notably, pro-inflammatory factors play an important role in distinguishing it from apoptosis (28). Results from the present study also revealed elevated pyroptosis and secretion of inflammatory factors in HK2 cells under high glucose environment. However, the mechanism underlying RTECs pyroptosis in DKD has not been fully understood. Previous studies have demonstrated that the NLRP3 inflammasome is not only the molecule most associated with pyroptosis, but can also be activated by diverse stimuli (29, 30). In fact, one study revealed its crucial role in pyroptosis initiation and pro-inflammatory cytokines production in DKD (31). Liu et al. (6) found that lncRNA MALAT1 could regulate RTECs pyroptosis by inhibiting miR-30c targeting for NLRP3 in DKD. This indicates that non-coding RNA may play an important role in regulating DKD RTECs pyroptosis. CircRNAs, a type of new non-coding RNAs that exist widely and stably in many species, have been implicated in regulation of gene expression and participating in the occurrence and development of various diseases (4). Previous studies have shown that circ_WBSCR17 could aggravate HG-induced HK-2 cell injuries by activating SOX6 (32), while circACTR2 regulated high glucose-induced pyroptosis, inflammation and fibrosis in RTECs (5). Therefore, we hypothesized that circRNAs may participate in the pyroptosis of DKD RTECs by regulating inflammasomes, although no study has reported this phenomenon. To confirm our conjecture, we analyzed patterns of circ_0004951 expression in kidney tissues from six DKD patients.

Our results revealed that circ_0004951 was significantly upregulated in HK2 cultured under HG conditions, while its knockdown not only suppressed pyroptosis but also downregulated expression of inflammatory factors, namely IL-18 and IL-1β. Thus, circ_000495 might be not only be involved in pyroptosis of DKD RTECs, but also play an important role in the occurrence and development of DKD. Next, we performed bioinformatics analysis and found that circ_0004951 can bind onto miR-93-5p. Previous studies have shown that miR-93-5p is markedly downregulated in mesangial and HK2 cells under HG environment (15, 18). Therefore, low miR-93-5p expression has been associated with pathogenesis of DKD. Subsequently, we conducted dual-luciferase reporter assay, and found that miR-93-5p was significantly upregulated following downregulation of circ_0004951 under HG environment, confirming that circ_0004951 could regulate miR-93-5p. Moreover, bioinformatics analysis results demonstrated that miR-93-5p could bind to NLRP3, a phenomenon that was corroborated by results from dual-luciferase reporter assay. Finally, results from rescue experiments revealed that down-regulation of miR-93-5p and upregulation of NLRP3 could attenuate the anti-pyroptosis and anti-inflammatory effects of circ_0004951 knockdown on HK2. Based on these, we concluded that circ_0004951 might be playing a role in RTECs pyroptosis of DKD by regulating miR-93-5p targeting NLRP3.

In conclusion, we provide the first report of circ_0004951's role in promoting inflammatory response and pyroptosis of RTECs in DKD patients *via* the miR-93-5p/NLRP3 inflammasome pathway. These findings have far-reaching implications in clinical diagnosis and treatment of DKD, although *in vivo* studies are needed to validate the observed physiological functions in patients.

## Data Availability Statement

The raw data supporting the conclusions of this article will be made available by the authors, without undue reservation.

## Ethics Statement

The studies involving human participants were reviewed and approved by Ethics Committee of The First Affiliated Hospital of Zhengzhou University (Zhengzhou, China). The patients/participants provided their written informed consent to participate in this study.

## Author Contributions

YW, LD, and LT: conceptualization. YW, LD, RW, YG, ZY, LY, and LW: investigation, methodology, data curation, and formal analysis. LT: funding acquisition. YW and LD: writing—original draft. YW, LD, RW, YG, ZY, LY, YL, and LT: writing—review and editing.

## Funding

This present study was supported by funds from the National Natural Science Foundation of China (Grant no. U1904134) and Henan Province Young and Middle-aged Health Science and Technology Innovative Talents (Leader) Project (Grant no. YXKC2020014).

## Conflict of Interest

The authors declare that the research was conducted in the absence of any commercial or financial relationships that could be construed as a potential conflict of interest.

## Publisher's Note

All claims expressed in this article are solely those of the authors and do not necessarily represent those of their affiliated organizations, or those of the publisher, the editors and the reviewers. Any product that may be evaluated in this article, or claim that may be made by its manufacturer, is not guaranteed or endorsed by the publisher.
